# Pulmonary artery sarcoma mimicking chronic pulmonary thromboembolism

**DOI:** 10.1590/0100-3984.2015.0046

**Published:** 2015

**Authors:** Marianna Nunes Batista, Miriam Menna Barreto, Renata Fukamati Cavaguti, Gláucia Zanetti, Edson Marchiori

**Affiliations:** 1 Universidade Federal do Rio de Janeiro (UFRJ), Rio de Janeiro, RJ, Brazil.

**Figure 1:**
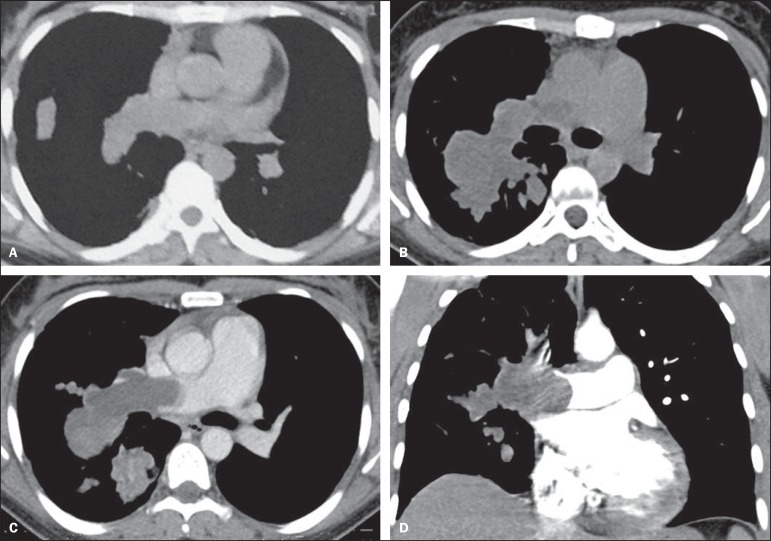
Axial chest computed tomography (**A**) demonstrating hypodense mass occupying the lumen of the right pulmonary artery. The luminal diameter is preserved. After seven months, follow-up with contrast-enhanced and non-contrast-enhanced axial (**B,C**) and coronal (**D**) computed tomography showed significant enlargement of the intraluminal mass determining dilatation of the affected vessels, with areas of contrast enhancement.

*Dear Editor*,

A 35-year-old woman was admitted in our institution with a 2-year history of dyspnea, hemoptysis and chest pain. Chest computed tomography (CT) demonstrated filling defects in the right pulmonary artery and some of its branches (Figure 1A). Transthoracic echocardiography showed right heart chambers enlargement and increased pulmonary artery systolic pressure. These test results associated to the patient's clinical history, allowed for the diagnosis of chronic pulmonary thromboembolism (PTE).

After six months of treatment without clinical improvement, a new contrast enhanced CT revealed a growing intraluminal filling defect and a lobulated mass on the right pulmonary artery and its branches, with areas of contrast enhancement (Figure 1 - B,C,D). In addition to the CT findings, magnetic resonance imaging identified restriction of water diffusion. These imaging findings yielded the diagnosis of pulmonary artery sarcoma (PAS).

A significant clinical worsening was observed and the patient died before she could be submitted to a diagnostic/therapeutic surgical procedure.

Vascular lesions of the chest have not been frequently described in the Brazilian radiological literature^(1-5)^. PAS is a rare malignant tumor that develops from mesenchymal cells in the intima of the pulmonary artery^(6)^. In general, it affects the central pulmonary arteries, close to the pulmonary valve^(7)^, resulting in significant morbidity and high mortality rates^(8)^. There is no predilection for sex, occurring most commonly in the fifth decade of life^(9)^.

In general, symptoms are nonspecific with dyspnea, cough, hemoptysis, chest pain and weight loss^(8)^, progressing to pulmonary hypertension, right ventricular failure, and possibly chronic *cor pulmonale*^(9)^. Clinical and radiological findings are frequently similar to thromboembolic disease. Due to its rarity and insidious growth pattern, PAS may be diagnosed as chronic PTE, leading to a diagnostic delay and inappropriate therapy such as anticoagulation or prolonged thrombolysis^(10)^.

At imaging studies, PAS presents as unilateral, intravascular lobulated masses with heterogeneous contrast enhancement, that may cause vascular distension and local extravascular dissemination^(11)^. Also, the lungs are frequently affected by metastases^(7)^. According to Yi et al.^(12)^, tomographic findings suggesting the diagnosis of PAS include low attenuation filling defect of the entire luminal diameter of a segment or of the whole extent of the main pulmonary artery, enlargement of the involved arteries and extraluminal extension of the tumor^(6,12)^. The prognosis is poor, with mean survival time of approximately one year and a half after symptoms onset^(8)^. Due to pulmonary artery occlusion and acute symptoms, surgical resection is generally the treatment of choice^(8)^.

In conclusion, the present case reinforces the important role of the imaging methods in the differentiation between pulmonary artery intimal sarcoma and chronic PTE. The relevant aspects for this differentiation, such as contrast enhancement, distention of the affect vessels and extraluminal extension, allow for a correct diagnosis, avoiding delay in the required surgical approach.
